# Resistance to the herbicides haloxyfop and iodosulfuron is common in commercial ryegrass (*Lolium*) seed lines

**DOI:** 10.1002/ps.8665

**Published:** 2025-01-20

**Authors:** Christopher E Buddenhagen, Zachary Ngow, Ben Wynne‐Jones, M. Philip Rolston

**Affiliations:** ^1^ AgResearch Ltd Hamilton New Zealand; ^2^ Better Border Biosecurity Research Collaboration (www.b3nz.org.nz) New Zealand; ^3^ Plant Health and Environment Laboratory Ministry for Primary Industries Auckland New Zealand; ^4^ Seed Industry Research Centre Christchurch New Zealand

**Keywords:** ACCase inhibitor, AHAS inhibitor, ALS inihibitor, EPSPS inhibitor, *Lolium* species, seed for sowing

## Abstract

**Background:**

Ryegrass (*Lolium* spp.) is a key forage providing a $14 billion contribution to New Zealand's gross domestic product (GDP). However, ryegrass can also act as a weed and evolve resistance to herbicides used for its control. Farmers suspected that imported seed might contribute to resistance issues. Herbicide resistance frequencies were investigated in commercial ryegrass seed lines intended for multiplication in New Zealand. Samples from 56 basic seed lots and 52 unique cultivars sourced from regions including New Zealand, United States, Europe and Japan were planted in field trials. Seedlings were then sprayed with three common herbicides: glyphosate, iodosulfuron, and haloxyfop. Surviving plants were retested to confirm resistance.

**Results:**

Resistance to haloxyfop and or iodosulfuron was detected in 79% of seed lines. However, frequencies were not significantly higher in imported lines (from United States and Europe) compared with New Zealand lines. Resistance was detected at frequencies between 0.00112% and 10% for haloxyfop and between 0.00212% and 14.28% for iodosulfuron Resistance to glyphosate was not found. There was no significant difference between the resistance detected in seed samples sourced from different seed companies.

**Conclusions:**

It was found that 63% of resistant lines had resistance frequencies rarer than 0.1%, but this is potentially problematic considering typical sowing rates. Imported *versus* domestic seed sources were not significantly different; they pose similar levels of resistance risk to farmers. *Lolium multiflorum* had a higher resistance frequency compared to *Lolium perenne* (although only six *L. multiflorum* lots were evaluated). Breeders should screen progeny of early crosses for herbicide resistance. © 2025 The Author(s). *Pest Management Science* published by John Wiley & Sons Ltd on behalf of Society of Chemical Industry.

## Graphical abstract

Eleven variety lines had resistance detected for iodosulfuron only, six to haloxyfop, and 28 to both herbicides. No seedlings were detected with resistance to glyphosate.

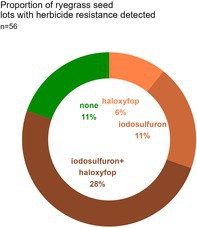

## 1 INTRODUCTION

Ryegrass (*Lolium* sp.) is a key forage crop species in New Zealand and is a key component of pastures which cover approximately 39% of the country. *Lolium* species are also globally important as forages and turfs. Ryegrass provides 14 billion New Zealand dollars (NZD) toward the country's gross domestic product (GDP) per annum (2012 estimate). The import, multiplication and export of ryegrass seed is vital to this and provided an estimated 80 million NZD in foreign revenue in 2021.^1^, ^2^, ^3^ New Zealand imports ryegrass seed from around 17 countries regularly for multiplication and re‐export; it is the fourth largest exporter of ryegrass seed globally.^4^ The top six trading partners for the crop are The Netherlands, United States, France, Australia, Denmark, and the United Kingdom.^5^ New Zealand had 42 direct trading partners for ryegrass seed, with those trading partners' own connections (indirect trading partners) bringing the network connected to New Zealand to up to 134 countries. While ryegrass species are very beneficial to agriculture, they are also well‐known weeds that have evolved herbicide resistance repeatedly, and at rates faster than many other weed species.^6^, ^7^, ^8^

Ryegrass seed is grown in crop rotation with wheat, barley and other arable crops. To grow certified seed in New Zealand, growers are required to have not grown any type of ryegrass in that field for at least the two previous seasons (though the same variety can be grown repetitively) and they must meet isolation requirements where no ryegrass can be found within specified distances, for example, 200 m.^9^ A challenge to these growers has been the increase in prevalence of herbicide resistant ryegrass in the Canterbury region since 2014.^7^, ^10^ Herbicide resistant ryegrass is found in other parts of the world that grow ryegrass seed, such as the United States, Denmark and Germany.^5^, ^6^ The contamination of ryegrass seed lines with herbicide resistance genes presents a risk of spreading resistance through global supply chains. Many arable farmers in New Zealand believe that their resistant ryegrass has come from the imported seed that they sowed. There is an implication that farmers believe that (1) herbicide resistance is rare in most local populations of ryegrass and (2) farming practices overseas somehow have led to higher rates of herbicide resistance than expected in New Zealand. These beliefs are shared by scientists to some extent. The number of species with herbicide resistance, and their prevalence, may be lower in New Zealand than in Australia, and some farming practices (e.g., frequent crop rotation) may explain the difference.^11^

Resistance‐conferring mutations in populations or alleles not previously exposed to herbicides are often assumed by weed scientists to be as low as 1:1 000 000 (0.0001%) or rarer^12^ but the initial frequencies are seldom estimated in the field.^13^ However, haloxyfop resistance in the New Zealand *Lolium perenne* cultivar ‘Nui’ was recently detected incidentally in a trial and estimated to occur at a frequency of 0.00133% (John Caradus unpublished data 2023). The most relevant and robust study on the topic is one from Australia, where rigid ryegrass (*Lolium rigidum* Gaudin) populations not previously exposed to herbicides had resistance rates between 0.001% and 0.012% for acetolactate synthase (ALS) inhibiting herbicides^14^ and for acetyl‐CoA carboxylase (ACCase) resistance frequencies ranged from 2.6% to 0.09%.^15^ In another study (not peer reviewed) the average frequencies of 1% × 10^−2^ were found for *L. rigidum* in pasture areas for diclofop‐methyl with no known history of Group 1 herbicide use.^16^ For a dicot weed *Amaranthus tuberculatus* (Moq.) J. D. Sauer wild populations were estimated to have lower frequencies of ALS target site genes conferring resistance compared with agricultural ones but frequencies still ranged from 2% to 22%.^17^ If *L. perenne* or *Lolium multiflorum* Lam. were planted out at the usual rate for the industry of 20 kg/ha (i.e., 10 000 000 seeds given a thousand weight of ≈ 2 g) and herbicide resistance rates were similar to the published Australian cases (*L. rigidum*), we might expect between 100 and 1200 resistant seeds per hectare for ALS or > 260 000 for ACCase inhibitors. There are no published studies examining the frequency of resistance in commercial *L. perenne* or *L. multiflorum* ryegrass cultivars.

We hypothesized that herbicide resistance frequencies in imported ryegrass lines would be higher than those in New Zealand lines, and that these rates would deviate from expected background levels seen in the few studies that addressed the issue in ryegrass unexposed to herbicides. To test this, we planted seeds sampled from various seed lots comprised of ‘breeders’ or ‘basic’ seed for the production of certified seed. Breeders seed is produced in small quantities and is the purest and most genetically stable seed of a particular variety. Breeders seed is used to produce basic seed and may be multiplied one or two generations to produce larger volumes, but in controlled conditions, and is expected to have sufficient varietal purity to achieve certification^9^ and be largely free of weed seed contaminants. Seedlings were treated with three herbicides registered for ryegrass control, all known to be linked to ryegrass resistance: glyphosate [this inhibits enolpyruvyl shikimate phosphate synthase (EPSPS); Group 9], iodosulfuron (an ALS inhibitor; Group 2), and haloxyfop (an ACCase inhibitor; Group 1).

## 2 METHODS

The field component of the study was conducted at the Foundation for Arable Research's Northern Crop Research Site (NCRS) in Tamahere, in the North Island, New Zealand. Field trials were done in late spring and summer of 2021 and 2022.

### 2.1 Plant materials

We sourced 0.5–1 kg samples of 56 ryegrass seed lots (52 cultivars) destined for seed multiplication, from New Zealand seed companies PGG Wrightson Seeds, Barenbrug, Luisetti, RAGT, and Grasslanz Technology Ltd. The samples included samples from seed lots of *L. perenne* (24 forage; 25 turf), six of *L. multiflorum* (five forage; one turf) and one *L. hybridum* (forage). Two of those samples were sourced from seed originating in Japan, 14 from United States, 28 from Europe and 12 from New Zealand. All the seed samples used in this study are ‘breeders’ or basic ‘basic’ seed. Cultivar names were anonymized here to protect the interests of the seed companies. The companies supplied the seed knowing what was intended, indicated their support for the study's goals, and have received their specific cultivar results with interest. The Seed Industry Research Centre and the Foundation for Arable Research staff represent both the seed industry and farmer groups and helped us to coordinate the provision of samples by seed company representatives.

### 2.2 Plot layout and sowing

Five samples of 20 seeds from each cultivar line were weighed to estimate the thousand seed weight for each seed lot. Seed from each seed lot were divided evenly by weight into 12 portions (four reps across three herbicide treatments) before planting–seeding rates were between 17 539 and 37 500 seeds per plot (2339 to 5000 seeds per m^2^ and 70 156 to 150 000 seeds per seed lot per herbicide).

The site for each of two trials, previously planted in maize, was cultivated in October of each year, and rested for 2 weeks before being sprayed with glyphosate [Crucial, Nufarm, Auckland, New Zealand, +0.1% Pulse; 600 g active ingredient (a.i.)/L glyphosate] 2160 g a.i./ha (sprayer conditions are described later for the main experiment). The field remained fallow for about 8 months after the first trial and then received the same field preparation steps prior to sowing in the second year. Upon inspection, before, during, and temporally between the trials, there was no germination of ryegrass in the unsown space between plots or between sown rows. A previous weed seedbank study on an adjacent maize plot at the same site did not record ryegrass as present in the seed bank.^18^

The two trials, sown on the same field on 23 November 2021 and 25 October 2022, respectively, were each laid out in a randomized block design for 28 cultivars by three herbicides by four blocks for a total of 336 plots (Supporting Information Figs S1 and S2). In each year 28 unique seed lots and cultivar combinations were sown into 5 m by 1.5 m plots spaced 1.5 m apart using a ten‐coulter drill (Duncan CUST‐USDP). After sowing in each year, the whole trial site was rolled to ensure good seed contact with the soil. The next day it was sprayed again with glyphosate Crucial® 1440 g a.i./ha, ethofumesate Nortron®, Bayer, Auckland, New Zealand 2000 g a.i./ha (500 g/L ethofumesate) and the surfactant Pulse 0.1%. Glyphosate controlled any remaining weeds, while Nortron suppresses summer grass (*Digitaria sanguinalis* (L.) Scop.) and *Poa annua* L. establishment (ryegrass is not impacted by ethofumesate).

### 2.3 Seedling counting before and after applying the herbicide treatments

Prior to spraying the herbicide treatment (details later), we estimated how many ryegrass seedlings of each sample had established, and therefore would be exposed to each herbicide treatment. Six 10 cm × 10 cm quadrats were placed randomly along the sown rows and seedlings were counted. For each cultivar the average seedling density across the four plots was used to estimate the total number of seedlings exposed to each herbicide. Three weeks after spraying, percentage survival was estimated across each plot. For glyphosate and haloxyfop, plants were usually clearly dead or alive and with few surviving plants green and dead plants being obviously browned off. If only a few plants were alive, we counted them and estimated the proportion surviving *versus* the initial seedling count estimate to obtain a percentage surviving. For iodosulfuron treatments some plants were yellowing, or brown, while others were green. We counted those yellowing and brown plants as dead. We decided against using the small random quadrats again after spraying because most plots had rare survivors, and these would have often been missed if we had used the same random sampling method. If many plants were alive, we used the averaged estimated proportion surviving as assessed by two observers for the whole plot.

### 2.4 Applying the herbicide treatment

To apply the herbicide treatments, we used a John Deere 1025R Compact Utility tractor, fitted with a 250 L capacity sprayer powered by a PTO driven pump regulated to 200 kPa (Udor 6010.24 Pressure Regulator) to deliver 200 L/ha through a 6 m boom with 12 TeeJet AIXR 11004 nozzles (mounted 500 mm above canopy) when driven at 5.56 km/h. Between treatments, the tank was fully rinsed and the lines flushed with water. Plots were sprayed with either glyphosate at 1440 g a.i./ha (Crucial® Nufarm) with the surfactant Pulse at 0.1%, iodosulfuron at 7.5 g a.i./ha (Hussar® Bayer Crop Science, Auckland, New Zealand) with the surfactant Partner at 1%, or haloxyfop at 260 g a.i./ha (Gallant® Ultra, Corteva, New Plymouth, New Zealand) with the adjuvant Uptake at 1%. These are the field recommended rates in New Zealand.

### 2.5 Collecting survivors and confirming their resistance

The purpose here was to assess if the survivors we found were genuinely resistant, or had survived for other reasons, such as emerging after treatments were applied. Depending on availability, up to ten healthy survivors per plot were collected (usually 2–5) from within the planted rows in a plot. If there were many survivors, plants were collected from separate rows or at distances from other collected plants. Plants were transplanted with soil from the field into pots partially filled with potting mix and kept moist in a temperature‐controlled (18–25 °C) glasshouse at Ruakura, North Island, New Zealand. Some plants died after transplanting and could not be tested. After plants fully recovered from transplanting and had produced several tillers, we split them into individual tillers, transplanted them, allowed them a week or two to establish, then cut them to a standard height of 4–5 cm, then allowed regrowth to a height of 6–8 cm. Tillers were then resprayed at label rates with their treatment herbicides (at the application rates mentioned earlier). Herbicide treatments were applied with a moving belt sprayer, fitted with a single TeeJet TT11002 flat fan nozzle at 200 kPa, positioned 440 mm above the top of the trays, and calibrated to apply a water rate of 200 L/ha. The moving belt was positioned in the central third of the fan area for even coverage. The surviving treated tillered plants were counted 3 weeks after spraying.

### 2.6 Calculating resistance frequencies

Our goal was to estimate the proportion of herbicide resistant plants for each seed lot in the field trials. We used the highest recommended label rate for ryegrass (usually perennial ryegrass) as a single ‘discriminating’ dose *sensu* Beckie *et al*.^19^ The estimated number of plants within the seed lot that were exposed to the treatment was estimated across the four replicates. The proportion surviving the treatment was estimated from the numbers of seedlings surviving in the field adjusted by the proportion of confirmed as resistant in the glasshouse (see tiller testing earlier).

### 2.7 Statistics

**Figure 1:**
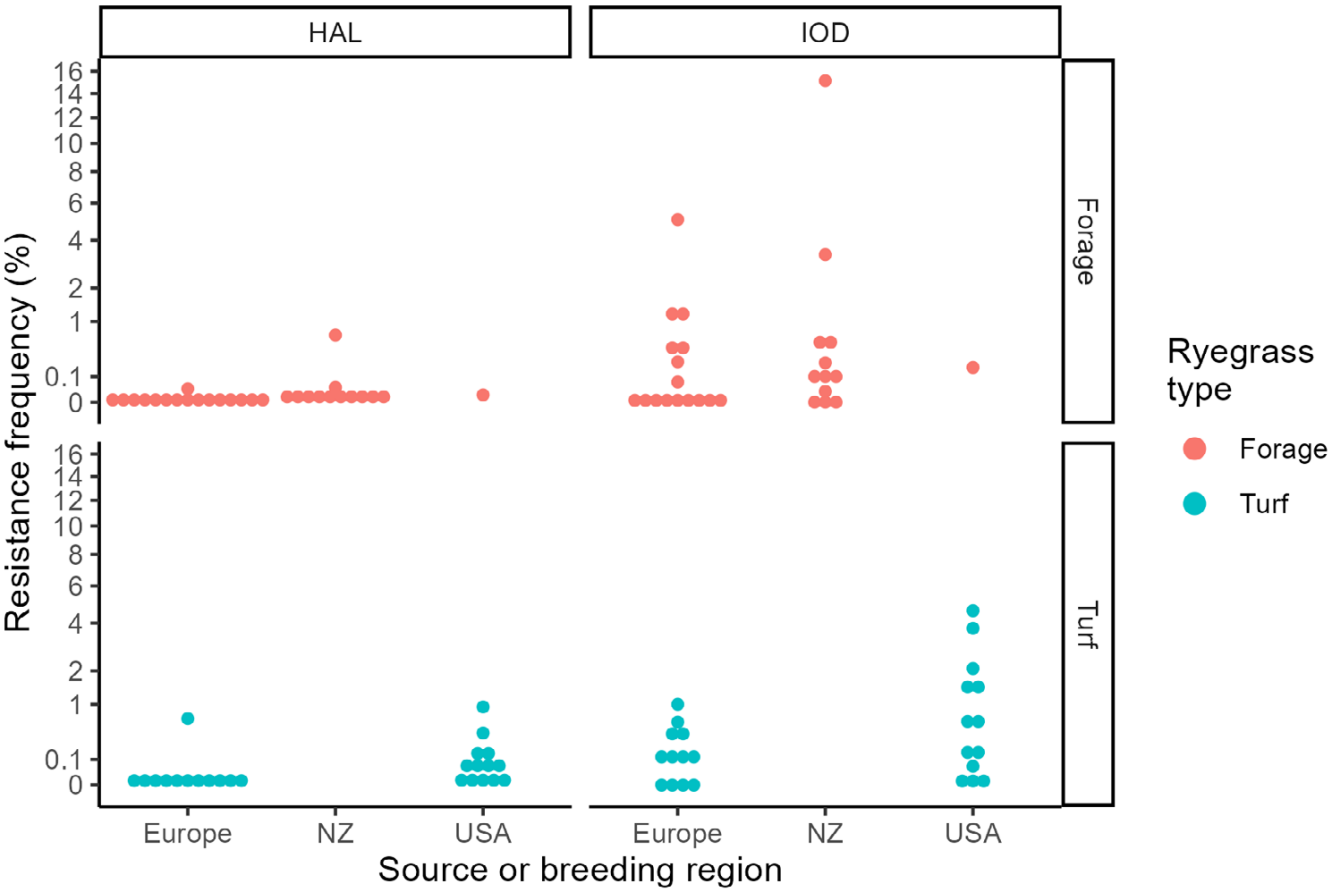
The frequency (%) of herbicide resistant seedlings detected in this study, with seed source or breeding region, plus treatment herbicides haloxyfop (HAL), and iodosulfuron (IOD) shown in panel columns and turf and forage types in panel rows. Two samples from Japan are omitted from this figure. The *y* axis ticks show the actual proportions on a transformed axis (arcsine square root) to distinguish differences between small proportions.

**Figure 2:**
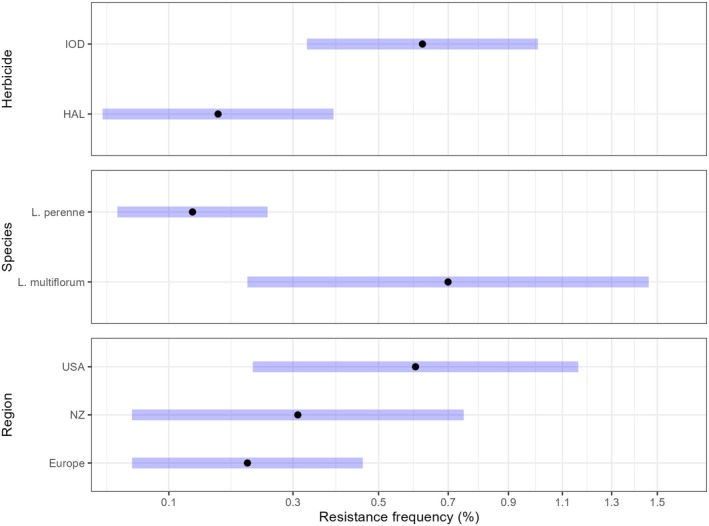
The estimated marginal means (black dots), and blue 95% confidence intervals of the frequency (%) of ryegrass seedlings that were resistant to iodosulfuron (IOD) and or haloxyfop (HAL) are shown for the factor combinations that were significant in the linear mixed model. The tick mark labels show the estimated proportions from the model, intervals vary to match the transformed (arcsine square root) scale, so small proportions can be shown. Two samples from Japan, and the only sample of *Lolium hybridum* were dropped from the model. [Correction added on January 23, 2025, after first online publication: Figure 2 caption has been updated.]

The frequency of resistant plants was graphed on ggplot^20^ using an arcsine square root axis transformation. That transformation was also used for variance‐stabilization^21^ before running a linear mixed model using the R function lmer,^22^, ^23^ with explanatory variables Herbicide, Species, turf_forage (describes the cultivar end‐use as a forage or turf), CompanyCode and Region and VarCode as a random effect (Supporting Information Data S1). No resistance to glyphosate was detected so all those data were excluded from the model. The two seed lot samples from Japan were also excluded because there were insufficient seed‐lot samples for inclusion as a separate region (no resistance was detected in these two lots). A tetraploid sample Var43, bred in New Zealand represented the only *Lolium* × *hybridum* Hausskn. example of the species so it had to be removed from the model. Expected marginal means were predicted from the model using emmeans^24^ and back transformed to proportions and presented graphically on the arcsine square root scale (Figs 1 and 2 and Table 1).

**Table 1:** Results of a linear mixed model fitting the arcsine square root proportion of herbicide‐resistant plants with explanatory factors as fixed effects (*P*‐values shown) and variety (VarCode) as a random effect. Back‐transformed means of resistant plant proportions (emmeans package) were converted to percentages with 95% confidence intervals and significance groupings (multcomp package). Means sharing a Significance group letter within a factor are not significantly different (*P* < 0.05). Glyphosate samples (no resistance) and samples from Japan or containing Lolium hybridum were excluded. Herbicides tested were haloxyfop (HAL) and iodosulfuron (IOD), species were L. perenne and L. multiflorum, with samples from five companies and three regions: USA, Europe, and New Zealand. SE = standard error.

Factor	Percentage resistant	SE	Lower 95% CL	Upper 95% CL	Significance group	*P*‐Value
Herbicide						0.000000496
HAL	0.166%	0.0877%	0.0371%	0.388%	a	
IOD	0.622%	0.1692%	0.3293%	1.007%	b	
Species						0.0147
*Lolium perenne*	0.130%	0.05%	0.0485%	0.250%	a	
*Lolium multiflorum*	0.699%	0.31%	0.2140%	1.460%	b	
Region						0.0833
Europe	0.214%	0.0987%	0.0613%	0.459%	a	
New Zealand	0.310%	0.1709%	0.0611%	0.749%	ab	
United States	0.602%	0.2332%	0.2235%	1.163%	b	
turf_forage						0.3537
Forage	0.293%	0.118%	0.103%	0.579%	a	
Turf	0.430%	0.166%	0.160%	0.830%	a	
CompanyCode						0.738
C1	0.255%	0.136%	0.05460%	0.603%	a	
C4	0.282%	0.125%	0.08590%	0.591%	a	
C2	0.351%	0.300%	0.00679%	1.212%	a	
C3	0.404%	0.212%	0.08993%	0.942%	a	
C5	0.530%	0.246%	0.14937%	1.141%	a	

## 3 RESULTS

Seedling emergence ranged from 49% ± 10% [standard deviation (SD); minimum = 33% and maximum = 70%] of seed sown in year 1 and 40% ± 8% (minimum = 25% and maximum = 64%) in year 2. As many as 90 000 or as few as 18 000 seedlings of each seed lot were exposed to each herbicide, but the median was 52 458 over trials in both years. Resistant seedlings surviving herbicide treatments with haloxyfop and or iodosulfuron were confirmed in 44 of the 56 individual seed lots and 44 of the 52 cultivars (79% and 85%, respectively). We collected 336 survivors from haloxyfop treated plots and 448 survivors from iodosulfuron treated plots, after growing them in the glasshouse and respraying them to confirm resistance mean tiller survival per sample was 44% ± 39% (SD) for haloxyfop and 37% ± 33% (SD) for iodosulfuron (eight and ten samples had no survivors for each herbicide, respectively). None of the 350 glyphosate ‘survivors’ were confirmed resistant after retesting. Note cultivar 43, with the highest frequency of resistance, was excluded from the statistical model as it was the only *Lolium* × *hybridum* Hausskn.; its resistance frequency was 14.29% (1:7 seeds). Twenty‐eight seed cultivar lines had frequencies of resistance to haloxyfop of less than 0.1% (1:1000), while only seven of the iodosulfuron‐resistant lines had frequencies of less than 0.1% (1:1000). The lowest *versus* highest frequencies we were able to detect for haloxyfop were 0.00112% (1:89500) *versus* 10% (1:10), and 0.00212% (1:47166) *versus* 14.29% (1:7) for iodosulfuron (Fig. S3). Eleven cultivar lines had resistance detected for iodosulfuron only, six to haloxyfop, and 28 to both herbicides. No seedlings were detected with resistance to glyphosate.

Some cultivars (but different seed lots) were tested once in year 1 and again in year 2, meaning that a total of 52 cultivars were tested from 56 unique seed lots. Where multiple seed lots from a cultivar were tested in different years the detected resistance varied. For cultivar (code = Var10) resistance was detected to haloxyfop and iodosulfuron in year 1 and only iodosulfuron in year 2, following the same format Var12 (haloxyfop and iodosulfuron; none), Var13 (haloxyfop and iodosulfuron; iodosulfuron) while Var14 (iodosulfuron and none; Data S1).

The forage and turf seed cultivars were from different source regions, for example none of the New Zealand cultivars were turf cultivars (Fig. 1). However, most regions showed a similar range of resistance levels within each herbicide (Fig. 1). The analysis of variance on the transformed proportions showed that the explanatory variables Herbicides and Species (*L. perenne*; *L. multiflorum*) were significant, but Region was marginal (Fig. 2 and Table 1). This is best understood by viewing the back‐transformed estimated marginal means comparisons (Fig. 2 and Table 1). Italian ryegrass *L. multiflorum* had higher frequencies of resistance than *L. perenne* (Fig. 2). For Region, New Zealand frequencies of resistance were intermediate between Europe and the United States which was significantly higher than Europe (Fig. 2). Explanatory variables such as the company code, and the end use type for each cultivar, that is, turf *versus* forage, were not significant (Table 1).

## 4 DISCUSSION

Our results confirmed farmers' suspicions that ryegrass seed for multiplication can be a source of herbicide resistant ryegrass, for Groups 1 and 2 (ACCase and ALS inhibitors) herbicides. We showed that 79% of the sampled seed lines contained herbicide resistant seeds. The frequency of resistance was significantly higher for Italian ryegrass than for perennial ryegrass, although only six samples of the former were evaluated. However, in opposition to farmers' expectations, the frequency of resistance in New Zealand seed lines was not the lowest, and was not significantly different to seed lines from Europe or the United States. The seeds lines from the United States had significantly higher frequencies of resistance compared with those from Europe. No seed company that provided seed was identified as having significantly higher frequencies of resistance than another. Therefore, there is evidence that local and overseas perennial ryegrass seed sources, or seed from any company can pose a risk to farming system effectiveness. However, no source region or company is significantly more risk prone. While Italian ryegrass cultivars pose a higher risk of herbicide resistance than growing perennial ryegrass cultivars, there was no difference in risk for turf *versus* forage seed lines (Table 1).

Resistance frequencies indicate that there is a risk of rapid evolution of resistance, given the amount of seed that is typically sown. Ten cultivar lines had resistance frequencies greater than 1% (1 × 10^−2^), while 30 of the resistant lines had frequencies less than 0.1% (1 × 10^−3^). The proportion of survivors in this study are similar to, and occasionally higher than, the published frequencies of heritable resistance seen in pristine (herbicide unexposed) rigid ryegrass populations described by Preston and Powles in Australia in 2002^14^ for ALS inhibitors; and generally rarer than those described by Neve and Powles for ACCase inhibitors.^15^ A tetraploid *Lolium hybridum* cultivar from New Zealand had the highest frequency of resistance to iodosulfuron at 15%, this was excluded from the statistical model because we had only one sample from this species. Even where no resistance was detected (for any of the herbicides) in our study, it is reasonable to assume that if we exposed enough seedlings from a seed line to the herbicides, we would eventually detect resistant individuals. Our use of a single discriminating dose (*sensu* Beckie *et al*.^19^) that is, the highest recommended label rate for *L. perenne* (260 g a.i./ha for haloxyfop, and 7.5 g a.i./ha for iodosulfuron) means we might not detect resistance levels otherwise detectable in dose response studies that use a range of lower rates. Though rare, the herbicide resistance frequencies for Groups 1 and 2 (ACCase and ALS inhibitors) herbicides that we detected could be problematic given that planting rates can be as high as 20 kg/ha (or 1000 seeds per m^2^). Thus, even low frequencies of resistance could cause problems in subsequent crop rotations and potentially promote the spread of the resistance traits into ryegrass populations in the vicinity. Farmers should assume that resistance occurs at low but meaningful frequencies in most ryegrass seed lines and that without a resistance management strategy that minimizes selection for the resistance, the problem could rapidly become worse under regular herbicide applications of Groups 1 and 2 herbicides.

More ryegrass lines had herbicide resistant plants detected for iodosulfuron (Group 2, ALS inhibitor) treatments, and the proportion resistant was also significantly higher than for haloxyfop (Group 1, ACCase inhibitor). However, 63% of the seed lots contained plants that were resistant to both groups. This is similar to the general pattern in arable farms in New Zealand, where ryegrass is often resistant to Group 1, Group 2, or both herbicide classes.^7^ That there was no glyphosate resistance in seed lines is reassuring. This also matches the pattern of arable farms,^7^ however, resistance was previously confirmed in a farmer supplied sample sourced from a barley crop,^25^ and there are glyphosate resistant ryegrass populations in New Zealand in vineyards.^8^

Mechanisms of resistance are not known for the cultivar lines we tested. We expect that both target site and or non‐target site cases will occur in individual plants and populations,^26^ or in this case, cultivar lines. In addition, for target‐site resistance, a number of different mutations would likely be detected, given that at least eight codon changes have been implicated in resistance to both ACCase, and ALS inhibitors, often multiple substitutions at each codon have been described.^27^ Investigating the mechanisms and genetics is potentially of interest, but beyond the scope of the current project.

The origins of resistance alleles in the commercial seed lots we studied remain uncertain. All the ryegrass species tested are obligate outcrossing species that can hybridise with each other. There is a distinct possibility that seed lots could be contaminated during the production process by nearby resistant ryegrass despite the precautions that are taken during seed multiplication to prevent this (see introduction). Resistance to ACCase and ALS inhibitors could stem from pre‐existing resistance‐conferring alleles maintained in the population prior to herbicide selection, as has been documented in blackgrass populations.^26^, ^28^ The samples with low resistance frequencies observed in this study could align with this concept, but higher frequencies such as a hybrid tetraploid cultivar we saw with a resistance frequency of 15%, suggest possible selection pressure from using herbicides or unintentional inclusion of resistant plants during breeding or multiplication. Further exploration of the population genetics of commercially propagated ryegrass populations could clarify the origins of the resistance alleles involved.^12^, ^29^

Nine of thirteen tetraploid cultivars had resistant seedlings detected in their seed lots. Because we did not carry out flow cytometry to test the ploidy of all the surviving plants, we cannot be sure if the resistant plants were also tetraploid. However, the source seed with the highest frequency of resistant plants 15% was from a tetraploid *L. hybridum* cultivar from New Zealand. This is the only case where we think a resistant parent was likely to have been included early in the breeding or multiplication process. There is little doubt that the resistant plants in this tetraploid *L. hybridum* cultivar are also tetraploid, given the high frequency of resistance. Additionally, the morphological differences between diploid and tetraploid plants and seeds should be sufficient to identify diploid contaminants and prevent the seed cultivar being certified. This contrasts with an Oregon study where no resistant tetraploid ryegrass populations were found.^30^ Sowing tetraploid ryegrasses in areas with herbicide‐resistant diploid ryegrass populations has been suggested as a strategy for limiting resistance by creating a reproductive barrier due to their different ploidy levels. However, this approach may not be effective if herbicide resistance is highly likely to evolve in tetraploid populations.^31^

In conclusion, frequencies of resistance to haloxyfop and iodosulfuron in commercial ryegrass seed lots were high for both species but higher still for *L. multiflorum* Lam. compared with *L. perenne*. The frequencies of resistance observed here would be impactful given the typical sowing rates we see in the field. Even a resistance frequency of 0.001% in ryegrass seed samples can, potentially, result in 100 resistant plants per hectare at typical sowing rates. For the samples taken from cultivar seed lines we obtained sourced from Europe, New Zealand, and the United States, there was no evidence that a geographic region or company has employed a management regime that leads to more resistance than another. More could be done to elucidate the exact level of resistance for all the cases detected here using dose response studies, the mechanisms of resistance, and the source of the problem (e.g., de novo mutations, contamination or standing variation). Given results from other studies, further genetic work would likely reveal a mix of target and non‐target site mechanisms, be that within each population, or individual plants.^32^, ^33^ However, from the perspective of farmers managing the problem, the initial frequency of resistance in their seed is potentially the most important consideration and warrants reporting here.

Our results have global implications for farmers sowing ryegrass, and for scientists studying resistance. It seems safer to assume most commercial seed lots, wherever they are sourced from, will contain resistant seeds at similar frequencies to those seen here, even if it is certified seed. If survivors are seen, weed managers should take steps to remove resistant plants. Ryegrass breeders and seed companies should screen for herbicide resistance early in the breeding and multiplication process. Where possible, seed companies could check their basic seed lines for resistance and should consider taking steps if seed lines have frequencies greater than 0.01%.

## CONFLICT OF INTEREST STATEMENT

The authors declare no conflict of interest.

## Supporting information


**Figure S1.** One of four blocks for the trial. Number plots were randomly assigned to 28 variety seed lots within the three herbicide treatment areas (colours). Herbicides were assigned randomly within blocks.
**Figure S2.** Good germination was achieved across the blocks.
**Figure S3.** Counts of variety lines with different rates of resistance for the two herbicides. The *x* axis is on an arcsine scale, but shows actual proportions on the tick marks. The herbicides are haloxyfop (HAL) and iodosulfuron (IOD).
**Code S1.** R code for the analysis of the data provided in Supporting Information Data S1.

**Data S1.** An excel spreadsheet of the data used in the analysis.

## Data Availability

The data that support the findings of this study are available on request from the corresponding author. The data are not publicly available due to privacy or ethical restrictions.
