# Supplementary material for: Resistance to the herbicides haloxyfop and iodosulfuron is common in commercial ryegrass (Lolium) seed lines
Source: Pest Manag Sci. 2025 Jan 20;81(6):2990–6. doi: 10.1002/ps.8665 (PMC12074625; doi:10.1002/ps.8665)
Supplement: Supplementary file 1 — Figure S1. One of four blocks for the trial. Number plots were randomly assigned to 28 variety seed lots within the three herbicide treatment areas (colours). Herbicides were assigned randomly within blocks. Figure S2. Good germination was achieved across the blocks. Figure S3. Counts of variety lines with different rates of resistance for the two herbicides. The x axis is on an arcsine scale, but shows actual proportions on the tick marks. The herbicides are haloxyfop (HAL) and iodosulfuron (IOD). Code S1. R code for the analysis of the data provided in Supporting Information Data S1. [file PS-81-2990-s002.docx]

# Supplemental materials


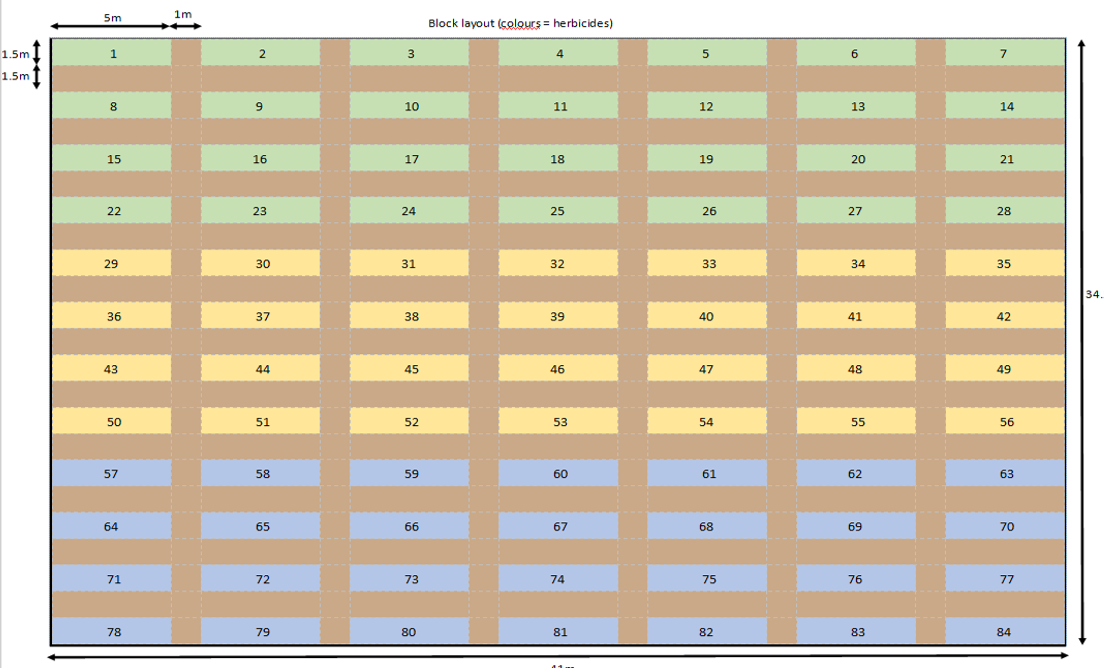


**Supplemental Figure S1)** One of four blocks for the trial. Number plots were randomly assigned to 28 variety seed lots within the three herbicide treatment areas (colours). Herbicides were assigned randomly within blocks.


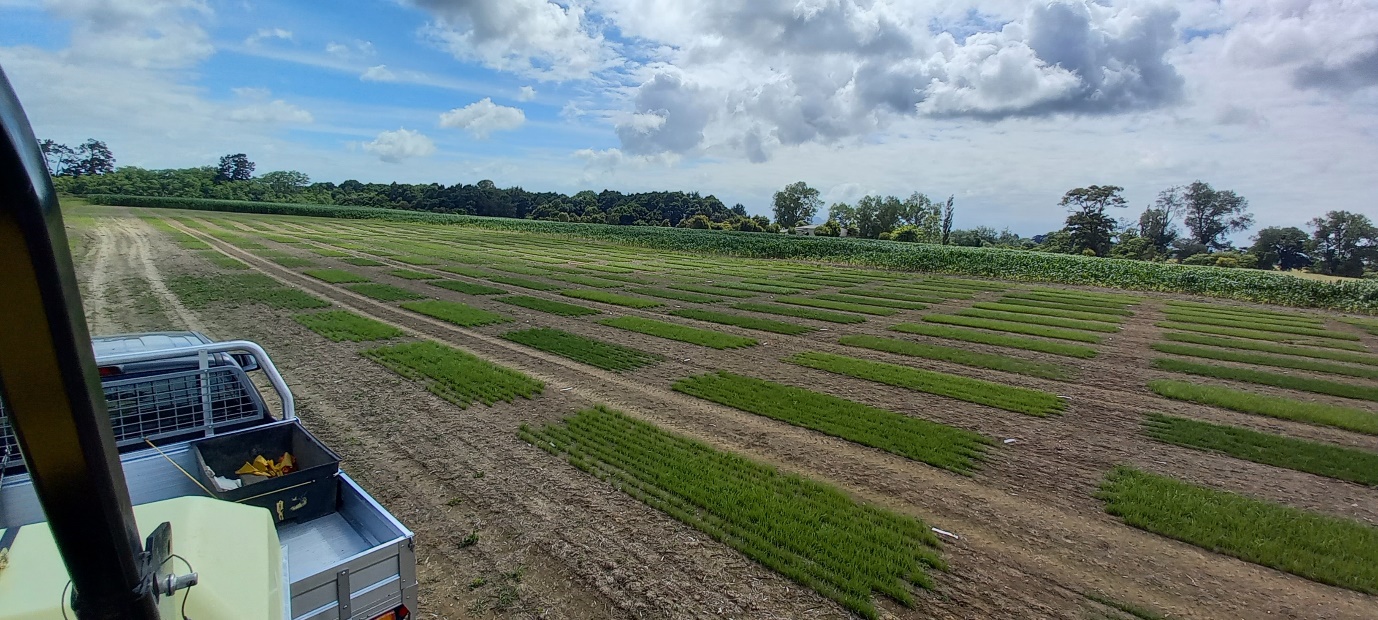


**Supplemental Figure S2)** Good germination was achieved across the blocks.


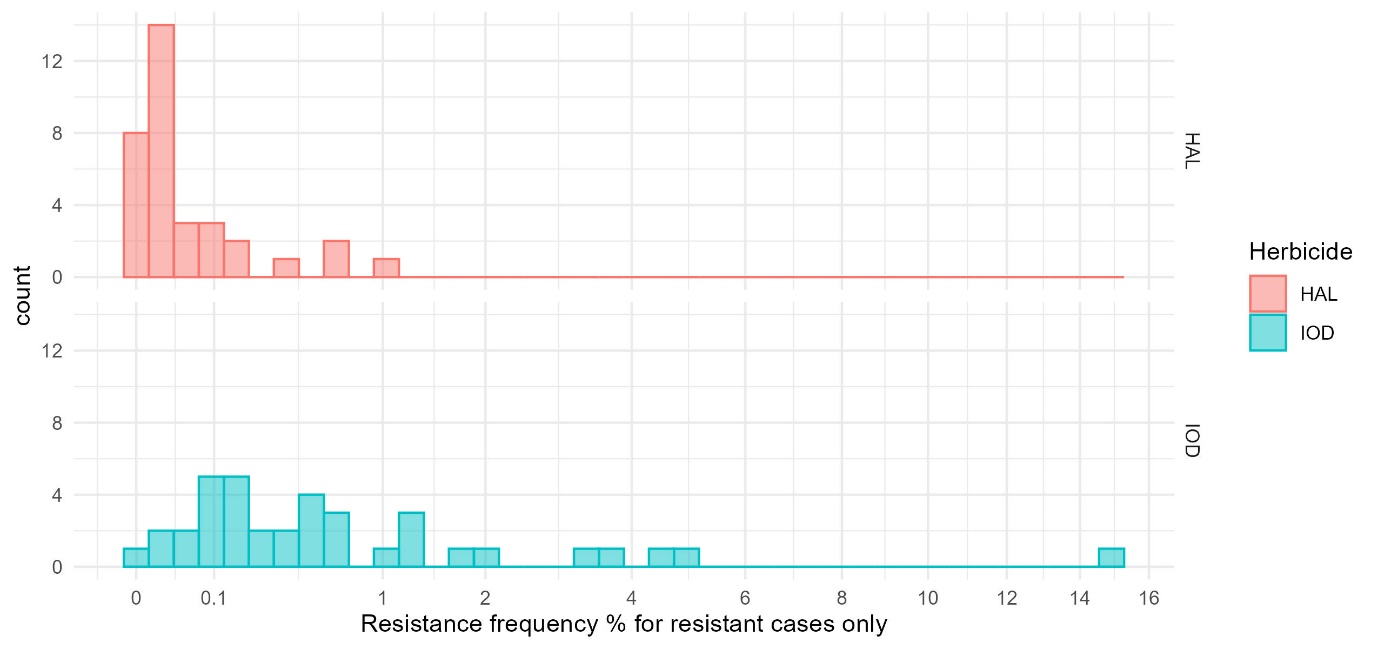


**Supplemental Figure S3)** Counts of variety lines with different rates of resistance for the two herbicides. The x axis is on an arcsine scale, but shows actual proportions on the tick marks. The herbicides are haloxyfop (HAL) and iodosulfuron (IOD).

**Supplementary data 1)** An excel spreadsheet of the data used in the analysis.

**Supplementary code 1)** R code for the analysis of the data provided in Supplementary data 1.

# Load required libraries

library(tidyverse) # For data manipulation and visualization

library(readxl) # For reading Excel files

library(clipr) # For copying data to the clipboard (if needed)

# Note: The multcomp package is needed but not loaded here, ensure it's installed.

# Set global options for better numerical output display

options(scipen = 100, digits = 6)

# Read the anonymized data from an Excel file

data <- read_xlsx("SimpleSheetForVarietyTrial2024vManuscript.xlsx", sheet = "Anonymised")

# Transform the dataset to long format for easier analysis

dat <- data |>

pivot_longer(

cols = starts_with("prop R"), # Columns with resistance proportions

names_to = "Herbicide", # New column to store herbicide names

names_prefix = "prop R ", # Prefix to remove from column names

values_to = "propR", # Column to store resistance proportions

values_drop_na = TRUE # Drop rows with NA values

)

# Retain only relevant columns and remove duplicate rows

dat <- dat |>

select(CompanyCode, VarCode, Species, ploidy, Region, turf_forage, Herbicide, Year, propR) |>

distinct()

# Display selected columns (for inspection/debugging)

dat |> select(CompanyCode, VarCode, Species, ploidy)

# Define a function for arcsine square root transformation

asinTransform <- function(p) { asin(sqrt(p)) }

# Apply the arcsine transformation to the resistance proportions

dat <- dat |> mutate(prop_asin = asinTransform(propR))

# Calculate percentiles for herbicides using the transformed data

# Back-transform the results to the original scale

outlier <- dat |>

filter(Herbicide != "GLY") |> # Exclude GLY herbicide

group_by(Herbicide) |>

reframe(

prop_asin = quantile(prop_asin, c(0.5, 0.75, 0.9, 0.95)), # Percentiles

quant = c(0.5, 0.75, 0.9, 0.95)

) |>

mutate(

back_trans = sin(prop_asin^2), # Back-transform to proportion

ratio_1_to_n = 1 / back_trans # Calculate 1:ratio

)

outlier # Display the results

# Calculate percentiles directly on proportions without transformation

dat |>

filter(Herbicide != "GLY") |>

group_by(Herbicide) |>

reframe(

propR = quantile(propR, c(0.5, 0.75, 0.9, 0.95)),

quant = c(0.5, 0.75, 0.9, 0.95)

) |>

mutate(ratio_1_to_n = 1 / propR)

# Percentiles grouped by herbicide and species

dat |>

filter(Herbicide != "GLY") |>

group_by(Herbicide, Species) |>

reframe(

propR = quantile(propR, c(0.5, 0.75, 0.9, 0.95)),

quant = c(0.5, 0.75, 0.9, 0.95)

) |>

mutate(ratio_1_to_n = 1 / propR)

# Visualize resistance proportions across regions and turf/forage types

dat |>

filter(Herbicide != "GLY", Region != "Japan") |> # Exclude Japan and GLY

ggplot(aes(x = factor(Region), y = propR, color = turf_forage, fill = turf_forage)) +

geom_dotplot(binaxis = "y", stackdir = "center") +

facet_grid(cols = vars(Herbicide), rows = vars(turf_forage)) +

xlab("Source or breeding region") +

ylab("Resistance frequency %") +

scale_y_continuous(

trans = "asn", # Arcsine scale

breaks = c(0, 0.001, 0.01, 0.02, 0.04, 0.06, 0.08, 0.1, 0.12, 0.14, 0.16),

labels = c("0", "0.1", "1", "2", "4", "6", "8", "10", "12", "14", "16")

) +

theme_classic() +

scale_fill_discrete(name = "Ryegrass \ntype") +

scale_color_discrete(name = "Ryegrass \ntype")

ggsave("Figure 1.jpg", width = 6, height = 4)

# Save breakpoints as character (e.g., for debugging or reuse)

dput(as.character(c(0, 0.001, 0.01, 0.02, 0.04, 0.06, 0.08, 0.1, 0.12, 0.14, 0.16) * 100))

# Create a histogram of resistance proportions by herbicide

dat |>

filter(Herbicide != "GLY") |> # Exclude GLY

filter(propR > 0) |> # Include only resistant cases

ggplot(aes(propR, colour = Herbicide, fill = Herbicide)) +

geom_histogram(bins = 40, alpha = 0.5) +

scale_x_continuous(

trans = "asn", # Arcsine scale

breaks = c(0, 0.001, 0.01, 0.02, 0.04, 0.06, 0.08, 0.1, 0.12, 0.14, 0.16),

labels = c("0", "0.1", "1", "2", "4", "6", "8", "10", "12", "14", "16")

) +

xlab("Resistance frequency % for resistant cases only") +

theme_minimal() +

facet_grid(rows = vars(Herbicide))

ggsave("Supplemental Figure.jpg", width = 8.5, height = 4)

# Load required libraries

library(lme4) # For linear mixed models

library(lmerTest) # For p-values in mixed models

library(emmeans) # For estimated marginal means (EMMs)

library(car) # For Type-II ANOVA

library(lattice) # For plotting random effects

library(multcomp) # For compact letter displays (CLDs)

library(ggplot2) # For additional plot customization

library(patchwork) # For combining multiple plots

# Filter data for the analysis

dat2 <- dat |>

filter(Herbicide != "GLY", Region != "Japan", Species != "L. hybridum") %>%

mutate(VarCode = factor(VarCode)) # Ensure VarCode is a factor

# Display levels of VarCode

levels(dat2$VarCode)

# Transformation function for arcsin(sqrt(p))

tran <- make.tran("asin.sqrt", 1)

# Build the linear mixed model

my.model <- with(tran,

lmer(linkfun(propR) ~ Herbicide + Species + turf_forage + CompanyCode + Region +

(1 | VarCode), data = dat2))

# Model summary and diagnostics

summary(my.model)

anova(my.model) # Type-I ANOVA

car::Anova(my.model) # Type-II ANOVA

hist(residuals(my.model)) # Residual diagnostics

lattice::dotplot(ranef(my.model, condVar = TRUE)) # Plot random effects

# Function to create plots and save them

create_plot <- function(my.model.emm, xlab_text, file_name) {

# Plot the estimated marginal means (EMMs)

plot_object <- plot(my.model.emm, type = "scale",

xlab = xlab_text,

breaks = seq(.001, 0.015, by = 0.002),

labels = c("0.1", "0.3", "0.5", "0.7", "0.9", "1.1", "1.3", "1.5")) +

coord_cartesian(xlim = c(0.0005, 0.016))

# Save the plot

ggsave(file_name, plot = plot_object, width = 5, height = 1.5, units = "in")

return(plot_object)

}

# Analyze and plot EMMs for each factor

plot_factors <- function(factor_name, xlab_text, file_name) {

my.model.emm <- emmeans(my.model, factor_name)

# Summary and compact letter display

summary(my.model.emm)

summary(my.model.emm, type = "response")

cld.my.model.emm <- cld(my.model.emm, alpha = 0.05, Letters = LETTERS,

adjust = "none", type = "response")

print(cld.my.model.emm)

# Generate and save the plot

create_plot(my.model.emm, xlab_text, file_name)

}

# Plotting for each variable

Herbicide_plot <- plot_factors("Herbicide", "Resistance frequency %",

"Herbicide_Backtransformed.png")

Species_plot <- plot_factors("Species", "Resistance frequency %",

"Species_Backtransformed.png")

Region_plot <- plot_factors("Region", "Resistance frequency %",

"Region_Backtransformed.png")

Turf_forage_plot <- plot_factors("turf_forage", "Proportion resistant",

"Turf_forage_Backtransformed.png")

CompanyCode_plot <- plot_factors("CompanyCode", "Proportion resistant",

"CompanyCode_Backtransformed.png")

# Combine all plots into a single figure using patchwork

final_plot <- (Herbicide_plot / Species_plot / Region_plot) *

theme_bw()+

plot_layout(axes = "collect")

# Save the combined figure

ggsave("Figure 2.jpg", plot = final_plot, width = 6, height = 4, units = "in")
